# Vaccinating to Protect a Vulnerable Subpopulation

**DOI:** 10.1371/journal.pmed.0040174

**Published:** 2007-05-22

**Authors:** Jonathan Dushoff, Joshua B Plotkin, Cecile Viboud, Lone Simonsen, Mark Miller, Mark Loeb, David J. D Earn

**Affiliations:** 1 Department of Ecology and Evolutionary Biology, Princeton University, Princeton, New Jersey, United States of America; 2 Fogarty International Center, National Institutes of Health, Bethesda, Maryland, United States of America; 3 Department of Biology, University of Pennsylvania, United States of America; 4 National Institute of Allergy and Infectious Diseases, National Institutes of Health, Bethesda Maryland, United States of America; 5 Department of Clinical Epidemiology and Biostatistics, McMaster University, Hamilton, Ontario, Canada; 6 Department of Mathematics and Statistics, McMaster University, Hamilton, Ontario, Canada; Harvard School of Public Health, United States of America

## Abstract

**Background:**

Epidemic influenza causes serious mortality and morbidity in temperate countries each winter. Research suggests that schoolchildren are critical in the spread of influenza virus, while the elderly and the very young are most vulnerable to the disease. Under these conditions, it is unclear how best to focus prevention efforts in order to protect the population. Here we investigate the question of how to protect a population against a disease when one group is particularly effective at spreading disease and another group is more vulnerable to the effects of the disease.

**Methods and Findings:**

We developed a simple mathematical model of an epidemic that includes assortative mixing between groups of hosts. We evaluate the impact of different vaccine allocation strategies across a wide range of parameter values. With this model we demonstrate that the optimal vaccination strategy is extremely sensitive to the assortativity of population mixing, as well as to the reproductive number of the disease in each group. Small differences in parameter values can change the best vaccination strategy from one focused on the most vulnerable individuals to one focused on the most transmissive individuals.

**Conclusions:**

Given the limited amount of information about relevant parameters, we suggest that changes in vaccination strategy, while potentially promising, should be approached with caution. In particular, we find that, while switching vaccine to more active groups may protect vulnerable groups in many cases, switching too much vaccine, or switching vaccine under slightly different conditions, may lead to large increases in disease in the vulnerable group. This outcome is more likely when vaccine limitation is stringent, when mixing is highly structured, or when transmission levels are high.

## Abstract 2

Jonathan Dushoff and colleagues model the benefits of different vaccination strategies and suggest that small differences in how populations mix can change the best vaccination strategy from one focused on the most vulnerable individuals to one focused on the most transmissive individuals.

## Editors' Summary

**Background.:**

Every winter, millions of people take to their beds with influenza—a viral infection of the nose, throat, and airways that is transmitted in airborne droplets released by coughing and sneezing. Most people who catch flu recover within a few days, but some develop serious complications such as pneumonia, and in the US alone, about 36,000 people—mainly infants, elderly, and chronically ill individuals—die every year. To minimize the morbidity (illness) and mortality (death) associated with seasonal (epidemic) influenza, the World Health Organization recommends that these vulnerable people be vaccinated against influenza every autumn. Annual vaccination is necessary because flu viruses continually make small changes to the viral proteins that the immune system recognizes.

**Why Was This Study Done?:**

Although infants and the elderly are particularly vulnerable to influenza, schoolchildren are more likely to spread the flu virus. Also, vaccination is more effective in schoolchildren than in elderly people. So could vaccination of schoolchildren be the best way to reduce influenza morbidity and mortality? Some Japanese and US data suggest that it might be, but policymakers need to know more about the likely effects of changing the current influenza vaccination strategy. They need to know in what circumstances the direct effects of vaccination (protection of vaccinated individuals from disease) outweigh its indirect effects (reduced infection in vulnerable individuals caused by the reduced spread of disease in the whole population) and when the opposite is true. In this study, the researchers have used mathematical modeling to investigate how vaccination affects the spread of diseases such as influenza for which a “core” group in the population spreads the disease and a distinct “vulnerable” group is sensitive to its effects.

**What Did the Researchers Do and Find?:**

The researchers developed a mathematical model in which members of each group mixed mainly with their own group (assortative mixing) and used it to predict how changing the proportion of a limited amount of vaccine given to each group might affect disease spread under different conditions. For example, they report that in a population in which the two groups were very unlikely to mix and viral transmission was low, switching vaccine from the vulnerable group to the core group initially increased infections in the vulnerable group because fewer individuals were directly protected but, as more vaccine was allocated to the core group, fewer vulnerable people became infected because the size of the epidemic decreased. When viral transmission was high, vaccination of the vulnerable group was always best. However, when viral transmission was moderate, shifting vaccine from the vulnerable group first increased, then decreased infections in this group before increasing them again. This last change occurred when vaccination in the vulnerable group was so low that viral transmission was sufficient to maintain the epidemic within this group.

**What Do These Findings Mean?:**

As with all mathematical modeling, the researchers' findings depend on the assumptions included in the model, many of which are based on limited information. The model also considers a population that contains only two groups, an unlikely situation in real life. Nevertheless, these findings indicate that in a population in which one group of people is mainly responsible for the spread of a disease and another is most vulnerable to its effects, the best vaccination strategy is very sensitive to how the groups mix and how well the disease spreads in each group. Small changes in these poorly understood parameters can change the optimal vaccination strategy from one that vaccinates vulnerable individuals to one that mainly vaccinates the people who spread the disease. Importantly, a beneficial change in strategy can become deleterious if taken too far, so policy makers need to approach potentially promising changes in vaccination policy cautiously. Finally, for influenza, the model supports the idea that using some vaccine stocks in schoolchildren might decrease morbidity and mortality among elderly people but suggests that—even if this turns out to be correct—if all the vaccine were given to schoolchildren, more old people might die. Thus, the most prudent policy would be to supplement rather than replace vaccination of the elderly with vaccination of children.

**Additional Information.:**

Please access these Web sites via the online version of this summary at http://dx.doi.org/10.1371/journal.pmed.0040174.

- US Centers for Disease Control and Prevention provide information about influenza for patients and professionals, including key facts about the flu vaccine (in English and Spanish)
- World Health Organization, fact sheet on influenza and information on vaccination (in English, Spanish, French, Arabic, Chinese and Russian)
- UK Health Protection Agency, information on seasonal influenza
- MedlinePlus encyclopedia entries on influenza and the influenza vaccine (in English and Spanish)
- Public disease mortality and morbidity data at the International Infectious Disease Data Archive (IIDDA)

## Introduction

Most influenza-associated deaths in the developed world occur in the elderly population, and current US vaccination policy gives highest priority to vaccination of persons at risk for influenza complications (primarily persons 65 y and older, but also children under age 2 y and those with chronic respiratory problems), and their contacts [1,2]. Recently, some authors have renewed suggestions that vaccinating schoolchildren, who respond well to vaccination and may have an important role in transmission in the population, could be an important component of a strategy to protect the whole population, including elderly people [3–8]. Inspired by questions arising from influenza policy, here we investigate the general question of the effects of vaccination in an infectious disease system in which the population has a “core” group that is particularly effective at spreading disease and is distinct from a “victim” or “vulnerable” group that is more vulnerable to the effects of disease (although not necessarily more susceptible to infection). This question has also been addressed by Patel and colleagues [9], who used genetic algorithms to find optimal vaccine strategies in a structured community model, and by Bansal and coworkers [10], who simulated a network population model. Both of these studies make detailed assumptions about population structure; our more general approach allows us to investigate the effects of varying mixing parameters.

To investigate vaccine strategies, we must distinguish between the direct effect of vaccination—protecting vaccinated individuals from contracting disease—and the indirect effect— protecting unvaccinated people by reducing the level of infectiousness in the population, and thus the risk of infection. We expect the direct benefit of vaccination to be greatest if we vaccinate the most vulnerable individuals, while the indirect benefit may be greatest if we vaccinate those individuals most active in transmitting infection. Thus, the best vaccine allocation strategy at the population level is not always obvious.

We used a simple model (see Methods) to illustrate some of the complexities that arise. Our model considers a population with two groups: a core group and a vulnerable group. We assume assortative mixing: individuals are most likely to mix with other individuals in the same group. For illustration purposes, we consider the question of how to allocate a fixed amount of vaccine between a more actively mixing population (e.g., schoolchildren) and a more vulnerable population (e.g. elderly). This question is directly relevant during a vaccine shortage (for example, the 2004–2005 influenza season [11]). It also illustrates the issues that arise from setting policy and prioritizing resource use, even when there is not an actual shortage of vaccine. We assume that vaccine is given before the influenza season begins, and that the effects last until the end of the season.

## Methods

We assume that the core and vulnerable groups differ only in their contact rate (extending this framework to differences in susceptibility to infection or tendency to transmit will produce qualitatively similar results). We implement assortativity using “preferred” mixing [12], meaning that people reserve a proportion of their contacts (the preferred mixing coefficient, or *p*) for their own group, and unreserved contacts are random (and may include additional intra-group contacts).

We expect the final size of the epidemic to be nearly independent of model details [13–15]. Epidemic size can be estimated by simulating differential equations or by numerically solving the final-size equations, which are straightforward, but which cannot be solved analytically [13]. Scripts to perform both sets of calculations using the free programming language R are available at http://algonquin.princeton.edu/vaccinealloc. Here we present results obtained by simulating each set of parameters for 30 disease generations using the simplest possible SIR model, starting from a prevalence of 10^−5^ per capita; results obtained by numerically solving the final-size equations are similar. The equations used for the SIR model are:

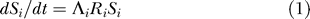


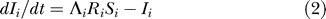
Here *S*_*i*_ and *I*_*i*_ are the proportion of the population represented by susceptible and infectious individuals in group *i*, and *t* is time rescaled in disease generations. Λ*_i_* is the proportion of group *i*'s contacts who are infectious, given by

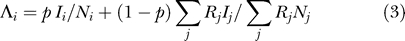
where *N*_*i*_ is the proportion of the population in group *i*, *R*_*i*_ is the subgroup reproductive number of group *i* [16] and *p* is the coefficient of preferred mixing.

## Results

**Figure 1:**
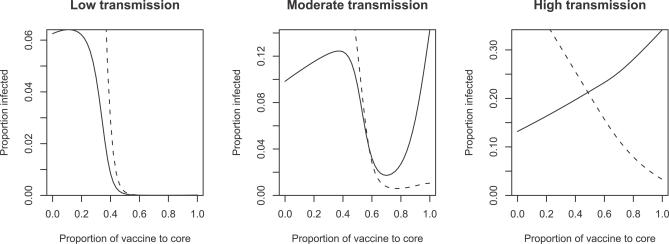
Complex Tradeoffs in Vaccine Allocation in a Population with Strong Assortative Mixing The proportion of “vulnerable” (solid lines), and “core” (dashed lines) individuals infected as a function of the proportion of vaccine given to core individuals, for three different transmission scenarios. In low transmission, the value of *R* for core and vulnerable individuals is 2.0 and 1.2, respectively; in medium transmission *R* is 2.4 and 1.6; in high transmission *R* is 2.8 and 2.0. The overall proportion of the population vaccinated is 0.5. The vaccine is assumed to have an efficacy of 80% in protecting core individuals and 50% in protecting vulnerable individuals. The two subpopulations are assumed to be of equal size. The coefficient of preferred mixing used is 0.7 (see Methods).

Figure 1 shows an illustrative example in a population with strongly assortative mixing (i.e., most mixing occurs within the groups). Inspired by recent evidence about influenza vaccines [17–22], we allowed the protective effect of the vaccine to be higher in the core than in the vulnerable group. Due to uncertainty about the effective size of both the vulnerable and core groups, we here set them equal: a more realistic model could explicitly include a third group representing healthy adults.

**Figure 2:**
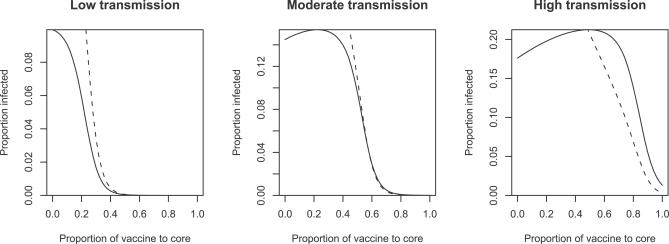
Vaccine Allocation Tradeoffs in a Population with Weak Assortative Mixing The proportion of “vulnerable” (solid lines), and “core” (dashed lines) individuals infected as a function of the proportion of vaccine given to core individuals, for three different transmission scenarios. Parameters are the same as in Figure 1, except that the coefficient of preferred mixing used is 0.1 (rather than 0.7).

We find that the effects of vaccine allocation in our example are complicated and sensitive to parameter values. When transmission is low, switching vaccine from the vulnerable group to the core group first increases incidence in the vulnerable group, since fewer individuals are directly protected. As more vaccine is allocated to the core group, however, a turning point is reached after which fewer vulnerable individuals are infected because the overall size of the epidemic is sharply reduced. This result is not surprising: in this case, vaccinating the vulnerable group provides substantial direct protection, while vaccinating a sufficient number in the core group achieves substantial (or even complete) indirect protection. In the high-transmission scenario, transmission within the vulnerable group is sufficiently important that it is always better to vaccinate this group (under the assumption that cases in the vulnerable group have much more severe consequences than those in the core group). This is true even though transmission rates in the core group are higher than in the vulnerable group, because mixing in this model is strongly assortative: individuals are most likely to infect others in the same group (compare to Figure 2).

With moderate transmission, the situation is more complicated: shifting vaccine from the vulnerable to the core group makes things first worse, then better, then worse again. The first transition is similar to the one seen in the low-transmission case: if a sufficient proportion of the core group is vaccinated the indirect protection gained outweighs the direct protection lost. The second transition also has an intuitive explanation: if too much vaccine is transferred away from the vulnerable group then a new kind of epidemic emerges in which the vulnerable group becomes “self-sufficient” in disease transmission [16] and can sustain its own epidemic.

We emphasize that these complexities are largely a result of our assumption of strong assortative mixing. Figure 2 shows results analogous to those of Figure 1, but in a population with weak assortative mixing (i.e., mixing is largely random across the population). We still see a tradeoff between direct and indirect protection, with the result that the worst allocation of vaccine, from the point of view of protecting the vulnerable group, is often intermediate between the two pure strategies of vaccinating in only one group, but we do not see the emergence of self-sufficient epidemics driven by transmission within the vulnerable group.

**Figure 3:**
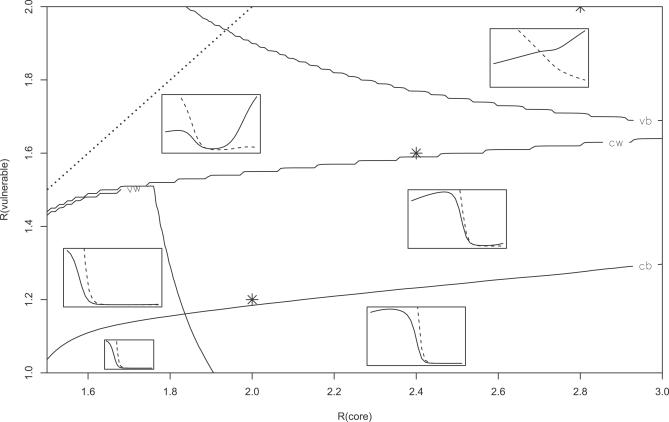
Parameter Regions with Different Qualitative Responses to Changes in Vaccine Allocation Insets show vaccine allocation tradeoffs at different points in parameter space, in the style of Figures 1 and 2. Contour lines (solid) separate regions in which the strategy that vaccinates only vulnerable individuals (v) or the one that vaccinates only core individuals (c) is or is not the best (b) or worst (w) on the curve describing changes in incidence in the vulnerable population. Two strategies on a given curve are deemed equivalent if they differ by less than 0.1% of the curve's maximum value. The large region in the middle right shows the case in which neither extreme strategy is best or worst; the extreme properties of any other region are described by the names of the line or lines separating the region from the middle right region. Asterisks correspond to the parameters shown in Figure 1. The dotted line shows values for which *R* is the same for both groups: our assumption that the core group transmits the disease more effectively than the vulnerable group does not hold in the region above this line.

We can assess a broader range of parameters by analyzing qualitative patterns shown in Figure 1 across a range of reproductive numbers. Figure 3 shows parameter domains in which we see six different shapes of the allocation response curve. These domains are divided by changes in whether the two pure strategies (vaccinate children first, or vaccinate elderly first) are best, worst, or intermediate in terms of the number of cases seen in the vulnerable group. This figure underscores the complexity of finding the right vaccination strategy in a structured population. The two large domains in the upper left and center right both have internal maxima and minima. In particular, this means that continuing to change in a direction that has made things better could make things worse, and conversely. It is also important to note that there is a wide range of parameters (the two upper regions) in which giving all the vaccine to the core group is the worst strategy for protecting the vulnerable group, despite the fact that there is higher vaccine efficacy in the core group, and the core group is also typically more active at transmitting the disease.

For the purposes of illustrating strategy tradeoffs in Figure 3, we have fixed the population-level vaccine coverage at 50%, and also fixed vaccine efficacy. It is worth noting, though, that the results are driven by the post-vaccination “effective” reproductive numbers in our two subgroups. Thus, we expect to see qualitatively similar results if we considered higher (or lower) levels of effective coverage, combined with higher (lower) reproductive numbers.

Two alternative versions of Figure 3 are shown in Figures S1 and S2. Because of concerns about vaccine efficacy in elderly people, for Figure S1 we repeated our analysis with all parameters the same, but using a vaccine efficacy of 30% instead of 50% for the vulnerable group. The results are largely similar. In particular, we still have large regions in which shifting some vaccine from vulnerable to core individuals improves protection of vulnerable individuals, while shifting all the vaccine makes things worse.

Because of the striking importance of assumptions about assortative mixing, we also repeated our analysis with all parameters the same, but the coefficient of preferred mixing set to 0.4, instead of 0.7 (see Figure S2). This change greatly reduces the size of the parameter regions in which shifting vaccine from the vulnerable to the core group has unwanted negative effects.

## Discussion

Influenza viruses are transmitted throughout communities, and some authors have suggested that vaccinating schoolchildren, who transmit influenza actively and respond well to vaccine, could be an important part of a strategy to protect more vulnerable groups [3–8]. There is evidence that vaccinating schoolchildren can protect other groups: from national-level patterns in Japan, which instituted and later repealed a policy of mandatory influenza vaccines for schoolchildren [4], and from two community-level field trials in the United States [23,24].

Here we asked what factors determine how best to allocate vaccination resources in a population that has distinct subgroups with different levels of transmission potential and vulnerability to serious morbidity from the disease. Our findings underscore the need for caution: because relevant parameters may be poorly known (e.g., details of how population mixing is structured) or may change from year to year (e.g., population immunity to the current dominant strain) it will be hard to predict in advance even the relevant qualitative regime for framing allocation questions. In particular, it is possible that a vulnerable group initially protected through population immunity of the core group may gradually accumulate susceptibility, increasing its effective reproductive number, *R*_i_. Thus a population could move, through time, from the parameter regime shown in the left graph of Figure 1 to the one shown in the center—and an initially effective control strategy (in this case, vaccinating the core group first) could become a disastrous one.

Despite the complexity of the question of optimal vaccine allocation, some general patterns can be seen in Figure 3. In particular, if transmission rates in both groups increase together, the relative value of giving vaccine to the elderly also increases. This is also true when we increase the transmission rate of the elderly alone. This simple pattern does not hold for the core group, however. In some cases, increasing the transmission rate of the core group results in a decrease in the amount of vaccine optimally given to this group, because indirect protection is relatively less effective than direct protection when transmission is high.

As we have shown, infectious disease dynamics are sensitive to the strength of assortative mixing in the population. We have illustrated possible scenarios using a model with only two groups, but real populations are far more complex in structure and behavior. Elderly individuals who live with extended families may show little or no mixing with other elderly individuals, while those who live in retirement communities or institutions may have very strong assortative mixing. These patterns may differ substantially across cultures: for example, grandparents may cohabit with children more frequently in Japan than in the United States. Such differences across populations are a further reason for approaching vaccine allocation decisions cautiously.

Vaccination policy is also sensitive to the relative efficacy of the vaccine in different groups. Recent work has raised important questions about the effectiveness of vaccine in very elderly people [21,22,25]. To the extent that vaccinating elderly persons is less efficacious at the individual level, it will also have less effect at the population level.

Influenza epidemics of a given subtype generally recur within a few years. Thus, the effective values of *R* for influenza are likely quite low, due to accumulation of cross-immunity in the population [26]. In other words, influenza disease parameters are likely similar to those shown in the lower left corner of Figure 3. Our analysis of disease dynamics in this parameter regime supports the argument that vaccination of children may be a good way to protect the elderly [3–5,7,8]. Nevertheless, our analysis also shows that the outcome of a vaccination policy is very sensitive to the details of disease transmissibility and to the structure of mixing within a population. Given the level of uncertainty about population structure—as well as the risk of an elderly-driven epidemic—prudent policy for influenza should focus on supplementing rather than replacing the vaccination of the elderly [5,8,25]. In contrast to annual epidemics, the value of *R* during an influenza pandemic—caused by the appearance of a novel subtype—could be higher than during an epidemic, although there is evidence that pandemic transmission has been low in the past [27–29]. For a pandemic, it will be hard to predict how the disease will be transmitted or who will be most vulnerable. Our results on the sensitivity of outcomes to basic disease parameters show that taking a dynamical approach could provide important insight in the debate over vaccination policy during an influenza pandemic [30,31].

## Supporting Information

Figure S1 Parameter Regions with Different Qualitative Responses to Changes in Vaccine Allocation, with Vaccine Efficacy in the Vulnerable Group Set to 30% Other parameters as in Figure 3. Contour lines (solid) separate regions in which the strategy that vaccinates only vulnerable individuals (v) or the one that vaccinates only core individuals (c) is or is not the best (b) or worst (w) on the curve describing changes in incidence in the vulnerable population. Two strategies on a given curve are deemed equivalent if they differ by less than 0.1% of the curve's maximum value.
(10 KB PDF)

Figure S2 Parameter Regions with Different Qualitative Responses to Changes in Vaccine Allocation, with the Preferred Mixing Parameter Set to 0.4 Other parameters as in Figure 3. Contour lines (solid) separate regions in which the strategy that vaccinates only vulnerable individuals (v) or the one that vaccinates only core individuals (c) is or is not the best (b) or worst (w) on the curve describing changes in incidence in the vulnerable population. Two strategies on a given curve are deemed equivalent if they differ by less than 0.1% of the curve's maximum value.
(10 KB PDF)
