# Supplementary material for: Vaccinating to Protect a Vulnerable Subpopulation
Source: PLoS Med. 2007 May 22;4(5):e174. doi: 10.1371/journal.pmed.0040174 (PMC1872043; doi:10.1371/journal.pmed.0040174)
Supplement: Supplementary file 2 [file pmed.0040174.sg002.pdf]

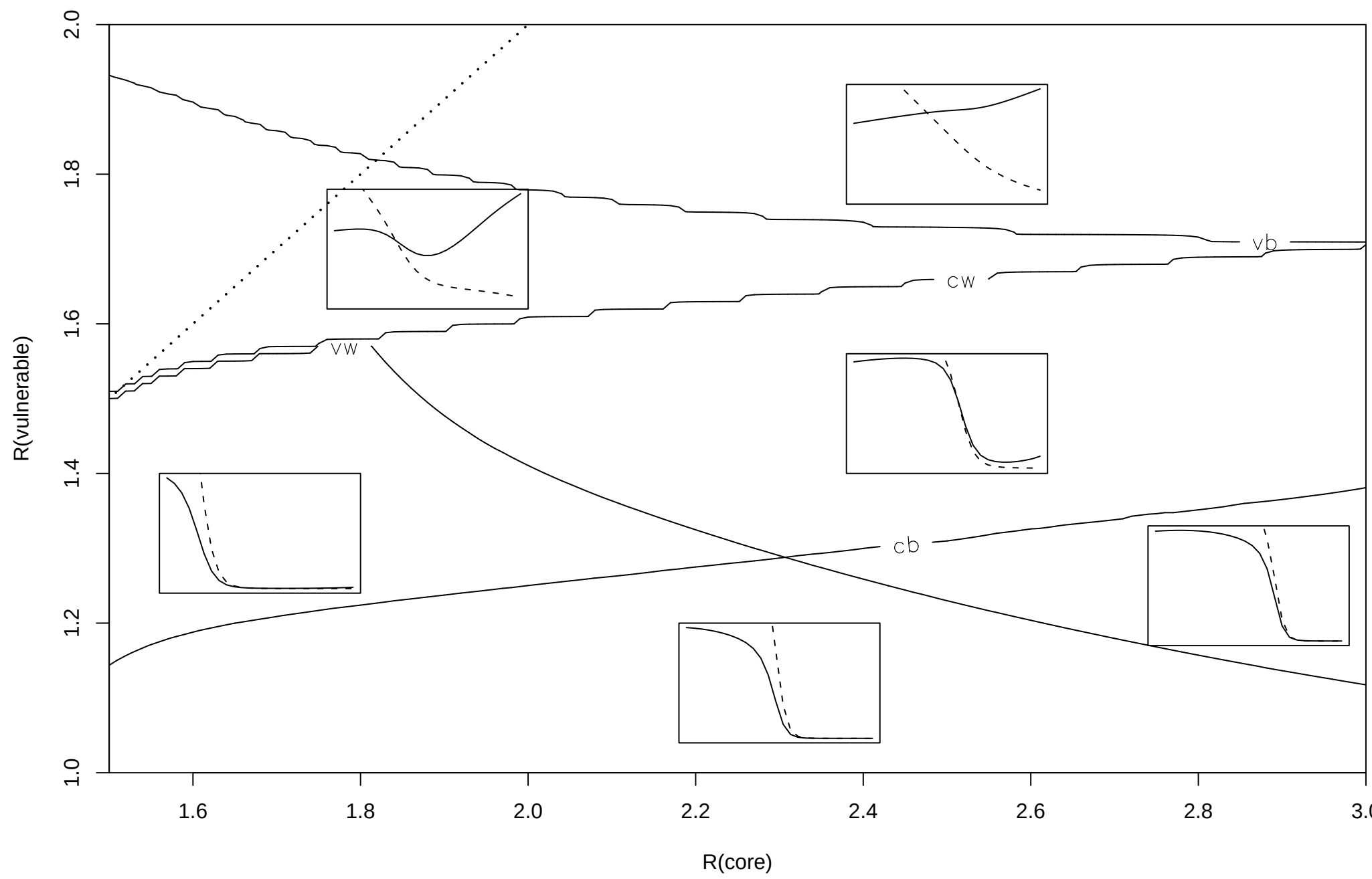

R(vulnerable)
R(core)
2.0
1.8
1.6
1.4
1.2
1.0
1.6
1.8
2.0
2.2
2.4
2.6
2.8
3.0
vb
cw
vw
cb
